# Hepatitis B virus X protein mediated suppression of miRNA-122 expression enhances hepatoblastoma cell proliferation through cyclin G1-p53 axis

**DOI:** 10.1186/s13027-016-0085-6

**Published:** 2016-08-15

**Authors:** Manikankana Bandopadhyay, Neelakshi Sarkar, Sibnarayan Datta, Dipanwita Das, Ananya Pal, Rajesh Panigrahi, Arup Banerjee, Chinmay K. Panda, Chandrima Das, Shekhar Chakrabarti, Runu Chakravarty

**Affiliations:** 1 ICMR Virus Unit, Kolkata, Indian Council of Medical Research, GB-4, 1st floor, ID & BG Hospital Campus, 57, Dr. S C Banerjee Road, Beliaghata, Kolkata, 700010 West Bengal India; 2 Molecular Virology Laboratory, Defense Research Laboratory (DRDO), Tezpur, Assam India; 3 Chittaranjan National Cancer Institute, 37, SP Mukherjee Road, Kolkata, India; 4 Saha Institute of Nuclear Physics, Bidhan nagar, Kolkata India; 5 National Institute of Cholera and Enteric Diseases, Kolkata, India; 6 Present Address: Department of Pathology & Lab Medicine, Tulane University School of Medicine, New Orleans, LA 70112 USA

**Keywords:** Hepatocellular carcinoma, HBx, miR-122, CCNG1, HepG2, HepG2.2.15

## Abstract

**Background:**

Hepatitis B virus (HBV) X protein (HBx) reported to be associated with pathogenesis of hepatocellular carcinoma (HCC) and miR-122 expression is down regulated in HCC. Previous studies reported miR-122 targets cyclin G1 (CCNG1) expression and this in turn abolishes p53-mediated inhibition of HBV replication. Here we investigated the involvement of HBx protein in the modulation of miR-122 expression in hepatoblastoma cells.

**Methods:**

Expression of miR-122 was measured in HepG2 cells transfected with HBx plasmid (HBx-HepG2), full length HBV genome (HBV-HepG2) and in constitutively HBV synthesizing HepG2.2.15 cells. CCNG1 mRNA (a direct target of miR-122) and protein expressions were also measured in both HBx-HepG2, HBV-HepG2 cells and in HepG2.2.15 cells. miR-122 expressions were analyzed in HBx-HepG2, HBV-HepG2 and in HepG2.2.15 cells after treatment with HBx mRNA specific siRNA. Expressions of p53 mRNA and protein which is negatively regulated by CCNG1 were analyzed in HBx transfected HepG2 cells; X silenced HBx-HepG2 cells and X silenced HepG2.2.15 cells. HBx induced cell proliferation in HepG2 cells was measured by cell proliferation assay. Flow cytometry was used to evaluate changes in cell cycle distribution. Expression of cell cycle markers were measured by real time PCR.

**Results:**

Expression of miR-122 was down regulated in HBx-HepG2, HBV-HepG2 and also in HepG2.2.15 cell line compared to control HepG2 cells. CCNG1 expression was found to be up regulated in HBx-HepG2, HBV-HepG2 cells and in HepG2.2.15 cells. Following siRNA mediated silencing of HBx expression; increased miR-122 levels were documented in HBx-HepG2, HBV-HepG2 and in HepG2.2.15 cells. HBx silencing in HBx-HepG2 and HepG2.2.15 cells also resulted in increased p53 expression. FACS analysis and assessment of expressions of cell cycle markers revealed HBx induced a release from G1/S arrest in HepG2 cells. Further, cell proliferation assay showed HBx promoted proliferation of HepG2 cell.

**Conclusion:**

Our study revealed that HBx induced down regulation of miR-122 expression that consequently increased CCNG1 expression. This subsequently caused cell proliferation and release from G1/S arrest in malignant hepatocytes. The study provides the potential to utilize the HBx-miR-122 interaction as a therapeutic target to limit the development of HBV related HCC.

## Background

Chronic Hepatitis B virus (HBV) infection serves as a major etiological factor for the development of hepatocellular carcinoma (HCC), a common malignancy worldwide. The X protein of HBV (also known as HBx) – an enigmatic and promiscuous viral oncoprotein is known to be crucially involved in the development of HCC. HBx alters host gene expression by activating various cytoplasmic signaling pathways (e.g., NF-kB, SRC, RAS, PI3K/AKT, JAK/STAT, SMAD and WNT) [1, 2]. HBx acts a transcriptional transactivator by interacting with nuclear transcription factors (e.g., CREB, ATF-2, OCT-1, TBP) and basal transcription factors [3] leading to increased cell proliferation and survival [4]. HBx modulates other cellular processes like reduction of DNA repair, impairment of p53-mediated apoptosis [5], activation of mitogen activated protein kinase (MAPK) pathways and induction of apoptosis by altering the TNFα and NF-kB signaling pathways [6–9]. HBx protein may increase the expression of TERT and telomerase activity, increasing the natural life of hepatocytes thus transforming to malignancies [10].

MicroRNAs (miRNAs) are a large family of functional transcripts in eukaryotic cells [11], which are small, endogenous, noncoding RNAs (21–23 nucleotides) that enhance mRNA degradation or inhibit posttranscriptional translation [12]. miR-122 –a liver specific miRNA exhibits key role in diverse aspects of hepatic function and pathogenesis [13, 14]. miR-122 are also found to be involved in neoplastic transformation and tumorigenicity [15–18]. It shows reduced expression in HCC and HCC-derived cell lines and culminates in hepatocarcinogenesis by targeting genes namely CCNG1, SRF, IGF1R, BCL2L2 and ADAM17 [18–20]. A recent study has revealed that chronic HBV infection is associated with loss of miR-122 expression, thus contributing to viral replication and carcinogenesis through CCNG1 modulated p53 activity. Further, HBV mRNA induced miR-122 down regulation has been shown to up regulate PTTG1- binding protein, which in turn also stimulates HCC tumor growth [21, 22].

Evidences are accumulating about the interaction between HBx protein and miRNAs. HBx protein and the HBx mRNA are known to act synergistically to repress miR-15a/16 expression in HepG2 cells through induction of c-Myc gene [23, 24]. miR-101 is shown to be down-regulated by the HBx protein and induces aberrant DNA methylation by targeting DNA methyl transferase 3A [25]. A previous study from our lab showed that HBx protein differentially modulates expression of miR-222, miR-21 and miR-145 in HepG2.2.15 cells [26].

As host cellular miRNA expressions are modulated by HBx protein evidenced in hepatic cancer cell line and chronic HBV infection reduces miR-122 expression, present study was performed to have an insight into the possible role of HBx protein of HBV in the modulation of miR-122 expression in hepatoblastoma cell line. We observed that expression of miR-122 were down- regulated in HepG2 cell line ectopically expressing HBx and 1.3 fold HBV genome through transient transfection and also in HepG2.2.15 cells. miR-122 expression was also reduced in LC and HCC patients infected with HBV. CCNG1- the target mRNA and protein of miR- 122, were modulated accordingly during transient transfection of HepG2 and in stable HBV producing cell line. miR-122 expression can be restored after HBx–siRNA treatment. Further, FACS analysis and cell proliferation assay showed HBx induced a release from G1/S arrest in HepG2 cells and promoted HepG2 cell proliferation.

## Methods

### Cell culture

The hepatoblastoma cell line HepG2 was maintained in Dulbecco’s modified Eagle medium (DMEM) with 10 % fetal bovine serum (Sigma Aldrich, Munich, Germany) at 37 °C in a humidified atmosphere with 5 % CO_2_. At approximately 80 % cell confluency, cells were harvested for RNA isolation. HepG2.2.15 cells, which are a kind gift of Dr. Tatsuo Kanda, Japan, were maintained in the RPMI1640 medium with 12 % fetal bovine serum in a 37 °C in a humidified atmosphere with 5 % CO_2_. The cells were generated every three days, and could be used when HBV DNA was detected stably in the supernatant.

### Study subjects

The patients recruited in this study were admitted to the SCB Medical College of Odisha, India from February 2013 to April 2014. A total of 102 advanced liver disease patients were recruited in this study which includes two groups: those with liver cirrhosis (LC) and those with hepatocellular carcinoma (HCC). These patients were screened for presence of HBV DNA and 57 were found to be HBV DNA positive. Finally 34 patients were selected having age group of 35–50 years. Age and sex matched 26 healthy individuals were enlisted as normal controls. The expression of miRNA- 122 was first compared between sera of advanced liver disease patients and healthy individuals (control). Subsequently advanced liver disease patients were subdivided to LC and HCC patients to indicate the significance of miRNA expression variation in these 2 distinct clinical groups.

The signed informed consent was obtained from all the study subjects and the study protocol was approved by Kalinga Gastroenterology Foundation, Odisha ethical committee. Patient samples were assigned on arbitrary identification based on the order of enrollment in our study. Study subjects were free of other viral infections, including human immunodeficiency virus (HIV), hepatitis C virus (HCV). Control samples were obtained from voluntary blood donors negative for HIV, HBV and HCV.

### Plasmids and RNA oligonucleotides

HBx plasmid -pCXN2-HBx was gifted by Dr. Tatsuo Kanda, Japan. 1.3 fold full length HBV genome cloned into pUC 19 vector was a gift of Dr. Mashashi Mizokami, Japan. HBx-siRNA [27] was used to produce small interfering RNAs (siRNAs) targeting HBx mRNA (Ambion, Texas, USA). siRNA duplexes with non-specific sequences were taken as negative control (NC) for siRNA experiments. siRNA transfection was carried out using Lipofecatmine 2000 (Invitrogen, Carlsbad, CA, USA) reagent and medium was replaced 6 h after transfection. RNA were extracted following different time interval.

### Cell transfection

Transfection was performed using Lipofecatmine 2000 (Invitrogen) following manufacturer’s instructions. Briefly, twenty four hours prior to transfection 5 × 10^5^ HepG2 cells were seeded into a six well plate. Cells were transfected with two doses - 1 μg and 2 μg of pCXN2-HBx plasmid, 1.3 fold HBV plasmid (puC19-1.3 HBV) and empty vector. In case of HBx and HBV plasmid transfection, after 48 h, cells were used for RNA extraction. For siRNA experiments RNA were extracted 48 h post transfection.

### RNA isolation from hepatoblastoma cells

Total RNA was extracted using Trizol reagent (Invitrogen) from 1 × 10^6^ ~ 2 × 10^6^ cells according to manufacturer’s protocol.

### cDNA synthesis and quantitative mRNA expression by real-time PCR

Reverse transcription was performed using the RevertAid first-strand cDNA synthesis kit following the manufacturer’s instructions (MBI Fermentas, Vilnius, Lithuania). RNA quantity and quality were assessed by determination of the optical density at 260 and 280 nm using spectrophotometry and additionally by visualization in agarose gels. Real-time PCR was performed in the ABI 7000 SDS (Applied Biosystems) using the Power SYBR Green (Applied Biosystems) according to the manufacturer’s instructions. The thermal cycling conditions comprised of 5 min incubation at 95 °C, followed by 40 cycles at 95 °C for 15 s, 60 °C for 30 s. All of the reactions were performed in triplicate. The relative quantity of the target mRNA was normalized to the level of the internal control GAPDH mRNA level. The relative quantitative analyses of the data were performed using SDS 7000 system software v1.2.3 (Applied Biosystems, USA). The relative quantitation of target gene expression was obtained using the comparative ΔΔC_T_ method [28].

### Western blot analysis

After 48 h of transfection, proteins were prepared for western blot analysis. Cells were washed in cold PBS and cellular proteins were extracted by using NP-40 buffer for 30 mins at 4 °C. Lysates were cleared by centrifugation and proteins were separated by gel electrophoresis. Membranes were blocked in TBS-0.1 % Tween 20 (TBS-T)/5 % (w/v) milk for 1 h at room temperature. Membranes were then incubated with primary antibodies diluted in TBS-T for 4 h at room temperature. Subsequently, membranes were washed with TBS-T and incubated with peroxidase-conjugated secondary antibody diluted in TBS-T at room temperature for 30 mins. Membranes were washed in TBS-T and bound antibodies were detected by enhanced chemiluminescence Reagents (Amersham Biosciences, Buckinghamshire, UK). Primary antibodies used were anti-cyclin G1, anti-p53 and anti-β-actin (Santa Cruz, USA). Proteins bands were quantified using a Densitometry scanner (Bio-Rad-GS-800, USA).

### miRNA assay

Approximately 35 ng of total RNA was reverse-transcribed in a 10-uL volume using the TaqMan miRNA reverse-transcriptase kit (Applied Biosystems, Foster City, CA) according to the manufacturer’s recommendations. Three microliters of the reverse-transcription reaction was used in each of the real-time PCR assays. Analyses of miR-122 were carried out in triplicates by means of the TaqMan human miRNA assays (Applied Biosystems) using SDS 7000 system software v1.2.3 (Applied Biosystems, USA).

### Cell proliferation assay

Cell proliferation was measured by Cell Titer 96 Aqueous One Solution Cell Proliferation Assay (Promega, USA), an MTT assay. Briefly, 3 × 10^3^ viable cells were counted in a hemocytometer using trypan blue. The cells were uniformly seeded in each well of 96-well plates and grown in 100 μl DMEM supplemented with 5 % FBS. After 24 h, when a semi confluent monolayer is obtained, HepG2 cells were transfected with HBx plasmid and empty vector; co transfected with HBx plasmid and HBx-siRNA. The plates were incubated at 37 °C in a humidified atmosphere of 5 % CO_2_ for another 48 h. Next, cells were suspended by trypsinization. Relative cell numbers were determined by incubating cells with MTT for 4 h. The resulting formazan was dissolved in DMSO and was measured at 490 nm in spectrophotometer (SpectraMax M2 Multi-Mode Microplate Reader). The absorbance at 490 nm is directly proportional to the number of viable cells. All experiments were performed in quadruplets.

### Cell cycle analysis

For cell cycle analysis, cells were harvested and thoroughly re-suspended in Phosphate Buffered Saline (PBS). The cells were then fixed by adding double volume of chilled 100 % ethanol (Merck), drop wise, with continuous vortexing. After incubating the mixture overnight at −20 °C, it was spun and the cells were re suspended in 500 μl of PBS. Cells were then incubated with RNaseA (0.2 mg/ml) for 30 mins followed by Propidium Iodide (50 μg/ml) (Sigma) at 37 °C for 1 h. Flow cytometric data acquisition was performed on the BD FACS Calibur platform.

### Statistical analysis

All statistical analyses were performed by using GraphPad Prism v.5.0 (GraphPad Software, USA). Data from three independent experiments were expressed as the mean ± SD. *T*-test (unpaired, two-tailed) was used for comparison. Probability levels of *P* < 0.001, *P* < 0.01 and *P* <0.05 were set for the determination of statistical significance.

## Results

### miR-122 expression is significantly decreased in transiently transfected and constitutively HBV producing hepatoblastoma cells and in HCC patients infected with HBV

**Fig. 1:**
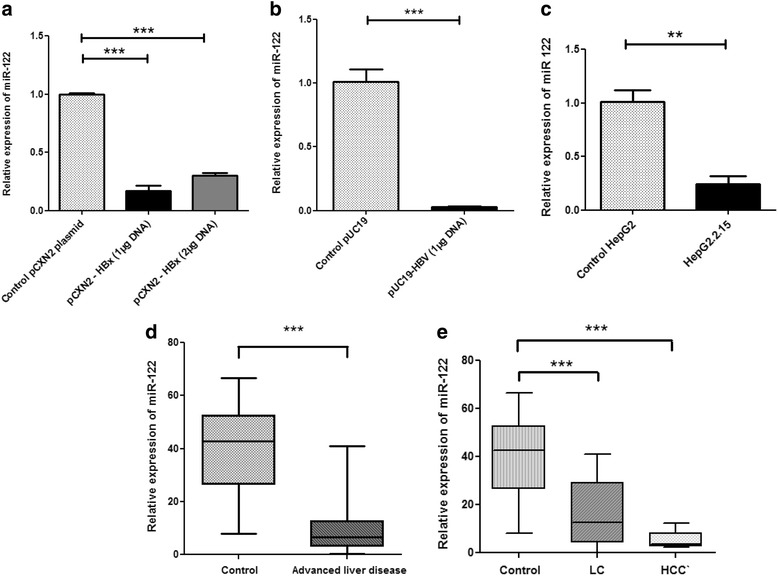
miR-122 expression is modulated by HBx in hepatoblastoma cells in vitro. **a** The relative expression of miR-122 in HepG2 cells transiently transfected with HBx expressing plasmid PCXN2-HBx or control vector. Cells are transfected with 1 μg and 2 μg of HBx plasmid respectively or pCXN2 as a control. **b** The relative expression of miR-122 in HepG2 cells transiently transfected with 1.3 fold full length HBV genome cloned into pUC19 plasmid or control vector. The cells are transfected with 1 μg pUC19-HBV or 1 μg pUC19. **c** The relative expression of miR-122 in constitutively HBV producing HepG2.2.15 cell line or control HepG2 cells. Cells were collected for analysis 48 h after each transfection. The miRNA expressions were measured by qRT-PCR. Plotted are the mean ± SD of three samples normalized to U6 expression (*P < 0.05, **P < 0.01, ***P < 0.001; Student’s *t*-test). **d** Real-time PCR analysis of miR-122 expression from patient serum samples. The miRNA levels in healthy controls were arbitrarily set as 1.0 and compared with advanced liver disease patients. **e** Comparison of miR-122 expression between healthy controls, LC and HCC patients. RNA was isolated from serum samples using miRVANA small RNA isolation assay and assayed using the TaqMan miRNA kit. The results were normalized to RNU6 endogenous control. Experiments were performed in triplicate. Error bars are means of ± standard deviation (SD). Mann–Whitney *U* test was performed to determine *P*-values (*P < 0.05, **P < 0.01, ***P < 0.001). LC = liver cirrhosis; HCC = Hepatocellular carcinoma

HepG2 cells were used for transient transfection to understand the possible impact of HBx on host miRNA expression. HepG2 cells were transfected with HBx plasmid (pCXN2-HBx) and 1.3 fold HBV genome (puC19-1.3 HBV). HepG2 cells were also transfected with empty expression vectors (pCXN2 and pUC19) and the expression pattern of miR-122 was measured in HBx transfected HepG2 cells. Interestingly, miR-122 was found to be significantly down regulated (*P* < 0.001) in HBx transfected cells, compared to the HepG2 cells transfected with empty expression vector (Fig. 1a). The expression was normalized by using RNU6 as internal control. The comparison of the fold changes measured during down regulation of miR-122 in HBx transfected HepG2 cells and in empty vector transfected HepG2 cells is presented in Table 1. Transfection with two different doses of HBx plasmid, however, did not exhibit any dose dependent down regulation.

**Table 1:** The fold changes (log2 values) during down regulation for miRNA- 122 in HBx transfected HepG2 cells compared to HepG2 cells transfected with empty expression vector

miRNA	HepG2 transfected with HBx plasmid (1 μg DNA)	HepG2 transfected with HBx plasmid (2 μg DNA)
miR-122	−0.17	−0.3

Transfection of HepG2 cells by 1.3 fold HBV genome, supported our previous experiments. Here also, we found significantly reduced (*P* < 0.001) levels of miR-122 expression in HBV transfected cells, compared to HepG2 cells transfected with empty pUC19 vector (Fig. 1b). When we measured the expression of this miRNA in HepG2.2.15 cells, which is a stable HBV producing cell line, we found that expression of mR-122 was significantly down regulated (*P* < 0.01) in comparison to the control HepG2 cells (Fig. 1c).

Next, miR-122 expression was analyzed in the sera of advanced liver disease patients infected with HBV. It was revealed that miR-122 expression was decreased significantly (*P* <0.001) in the sera of advanced liver disease patients when these patients were compared with healthy controls (Fig. 1d). This reduced expression of miR-122 was reflected in both LC and HCC patient groups when these two groups were compared separately with healthy controls (Fig. 1e). Interestingly, the comparison indicated that the HCC patients had lower miR-122 expression (*P* <0.001) than LC patients.

### Expression of target gene at mRNA and protein level due to transient transfection by HBx and in stable HBV producing cell

**Fig. 2:**
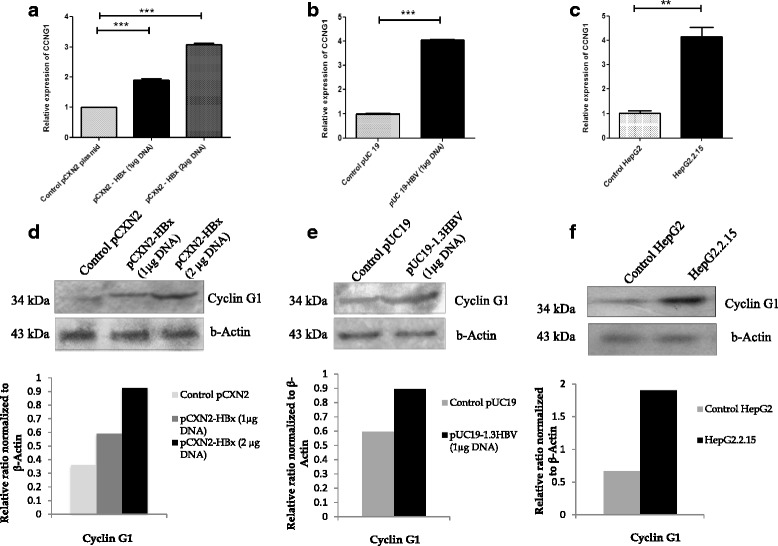
HBx modulated expression of target mRNA and protein CCNG1 (cyclin G1) in hepatoblastoma cells. **a** Relative expression of CCNG1 mRNA– target of miR-122 in HBx transfected HepG2 cells. Cells are transfected with 1 μg and 2 μg of pCXN2-HBx respectively or pCXN2 as a control. **b** Relative expression of CCNG1 mRNA in 1.3 fold full length HBV genome transfected HepG2 cells. Cells are transfected with 1 μg pUC19-HBV or 1 μg pUC19 control vector. **c** Relative expression of CCNG1 mRNA in HepG2.2.15 cell line and in control HepG2 cells. RNA were extracted 48 h post transfection. The mRNA expressions were measured by qRT-PCR and the expressions were normalized to GAPDH. Data are expressed as the mean ± SD from three independent experiments (*P < 0.05, **P < 0.01, ***P < 0.001; Student’s *t*-test). **d** Western blot confirmed protein cyclin G1 was increased accordingly in HBx transfected HepG2 cells. HepG2 cells are transfected with 1 μg and 2 μg of pCXN2-HBx respectively or empty pCXN2 as a control. **e** Expression of cyclin G1 protein in 1.3 fold HBV transfected HepG2 cells. HepG2 cells are transfected with 1 μg pUC19-HBV or 1 μg pUC19 vector as a control. **f** Expression of cyclin G1 in constitutively HBV producing HepG2.2.15 and control HepG2 cell line. Cells were collected for protein analysis 48 h after each transfection. β actin was taken as endogenous control. Protein bands were quantified using Densitometric scanner (Bio-Rad). Below are the graphical representations of intensity ratio between target protein cyclin G1 and β actin in each lane

Transfection of HepG2 cells by HBx caused up regulation of target mRNA CCNG1 expression compared to control cell line, i.e. transfected with empty expression vector (Fig. 2a). Transfection of HepG2 cells with 1.3 fold HBV genome revealed the same result as we observed in HBx transfected HepG2 cells. CCNG1 was found up regulated in 1.3 fold HBV genome transfected HepG2 cells when compared with HepG2 cells transfected with empty pUC19 vector (Fig. 2b). In both the cases (Fig. 2a, b) the up regulations of CCNG1 mRNA were significant (*P* <0.001). In case of HepG2.2.15 cell line, the CCNG1 expression was significantly elevated (*P* < 0.01) as compared to the control HepG2 cells (Fig. 2c). Expression of GAPDH was measured as internal control.

Western blot analysis confirmed that transfection by HBx caused up regulation of the target protein i.e. CCNG1 expression in HepG2 cells compared to control cell line. (Fig. 2d). When we transfected HepG2 cells with full length HBV genome, CCNG1 expression was found to be higher in HBV transfected cells than empty vector transfected HepG2 cells (Fig. 2e). Further, we compared target protein expression in HepG2.2.15 cell line and its control cell HepG2. We observed that CCNG1 was overexpressed in HepG2.2.15 cells in comparison to control HepG2 cells (Fig. 2f). This result was consistent with experimental results observed with miRNA expression in HBx or HBV transfected HepG2 cells. Expression of β-actin was considered as endogenous control in these experiments.

### HBx modulated miR-122 expression as revealed by RNA interference study

**Fig. 3:**
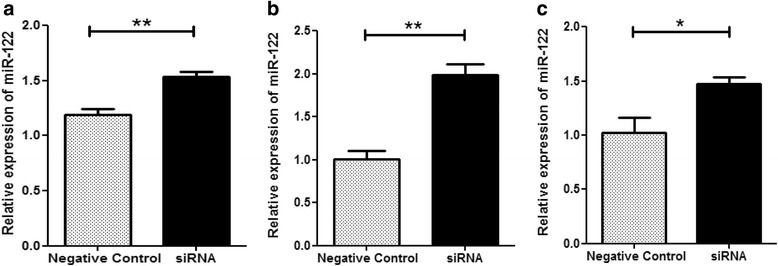
Restoration of miRNA expression after RNA interference study. **a** Relative expression of miR-122 in HepG2 cells by co-transfection with HBx plasmid (0.8 μg) and HBx mRNA specific siRNA (40 p mole) or Negative Control. **b** Relative expressions of miR-122 was measured when HepG2 cells were co-transfected with 1.3 fold full length HBV genome (0.8 μg) and HBx mRNA specific siRNA (40 p mole) or Negative Control. **c** HepG2.2.15 cells were transfected with HBx siRNA (80 p mole) or Negative Control and relative expressions of miR-122 was measured. Data plotted are the mean ± SD normalized to RNU6 expression. The experiments were performed in triplicate (*P < 0.05, **P < 0.01, ***P < 0.001; Student’s *t*-test). RNA were collected 48 h after each transfection

HepG2 cells were co transfected with HBx plasmid; 1.3 fold full length HBV genome and HBV X gene specific siRNA to knock down the HBx mRNA. HepG2.2.15 cells were also transfected with HBx siRNA. RNA was extracted at 48 h, post siRNA treatment. Both in HBx transfected and 1.3 fold HBV transfected HepG2 cells, significantly elevated expression (*P* < 0.01) of miR-122 was observed in 48 h post siRNA treatment (Fig. 3a, b). In HepG2.2.15 cell line, after 48 h post transfection, expression of miR-122 was reestablished (*P* < 0.05) (Fig. 3c).

### Silencing of HBx increases p53 mRNA and protein level in HBx transfected HepG2 and HepG2.2.15 cell line

**Fig. 4:**
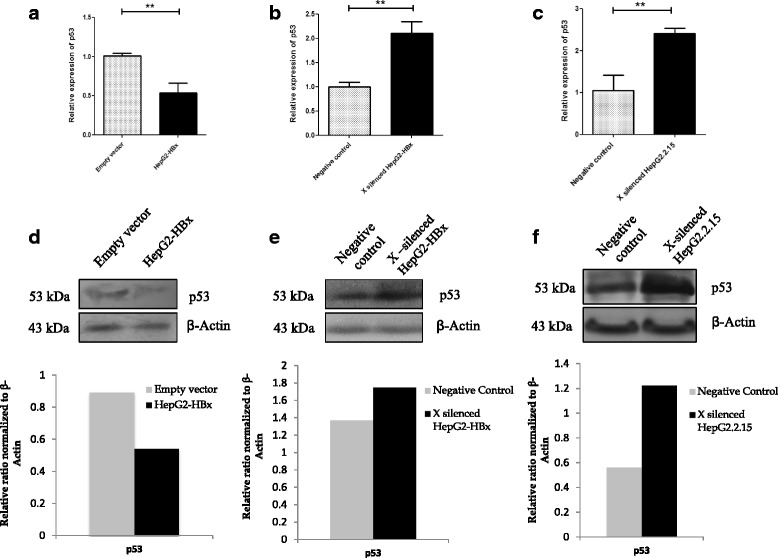
p53 mRNA and protein expression in HBx transfected HepG2 and HepG2.2.15 cells after RNAi treatment. **a** Relative expression of p53 in HepG2 cells after transfection with HBx expressing plasmid pCXN2-HBx or control vector. Cells are transfected with 1 μg of HBx plasmid or pCXN2 as a control. RNA were collected 48 h after each transfection. **b** Relative expression of p53 in HepG2 cells by co-transfection with HBx plasmid (0.8 μg) and HBx mRNA specific siRNA (40 p mole) or Negative Control. RNA were collected 48 h after each transfection. **c** HepG2.2.15 cells were transfected with HBx siRNA (80 p mole) or Negative Control and relative expressions of p53 was measured. RNA were collected 24 h post transfection. Data plotted are the mean ± SD normalized to GAPDH expression. The experiments were performed in triplicate (*P < 0.05, **P < 0.01, ***P < 0.001; Student’s *t*-test). **d** Corresponding p53 protein expression was analysed by western blot in HepG2 cells transfected with 1 μg of HBx plasmid or empty vector as a control. **e** p53 protein expression was analysed in HBx silenced HepG2 cells transfected with HBx plasmid. HepG2 cells were co-transfected with HBx plasmid (0.8 μg) and HBx mRNA specific siRNA (40 p mole) or Negative Control **f** Relative Expression of p53 protein was measured in HepG2.2.15 cells after siRNA treatment. HepG2.2.15 cells were transfected with HBx siRNA (80 p mole) or Negative Control. Cells were collected for protein analysis 48 h after each transfection. For HepG2.2.1.5, cells were collected at 24 h post transfection. β actin was taken as endogenous control. Protein bands were quantified using Densitometric scanner (Bio-Rad). Below are the graphical representations of intensity ratio between p53 and β actin in each lane

miR-122 increases p53 protein levels and activity through its negative regulation of CCNG1. Further, from our experiments we observed that HBx suppressed miR-122 expression and consequently up regulated expression of CCNG1. These two observations were very likely interrelated. As such, we decided to assess the modulation of p53 mRNA and protein level in HBx transfected HepG2 and HepG2.2.15 cells after knockdown of HBx. We observed that p53 mRNA and protein levels were reduced significantly in HBx transfected HepG2 cells (*P* < 0.01) (Fig. 4a, d). Interestingly, HBx knockdown in HBx transfected HepG2 and HepG2.2.15 cells resulted in increased expression of p53 mRNA and protein compared to their negative controls (*P* < 0.01) (Fig. 4b, e, cf respectively).

### HBx induces cell proliferation through modulation of miR-122 and CCNG1 interaction

We noted that HBx transfected HepG2 cells exhibited increased CCNG1 expression accompanied by a decrease of p53 mRNA and protein expression. Since CCNG1 has a role in cellular growth control, these results prompted us to verify involvement of HBx protein in cell proliferation and subsequent changes in the cell cycle through HBx induced modulation of miR-122–CCNG1 interaction.

**Fig. 5:**
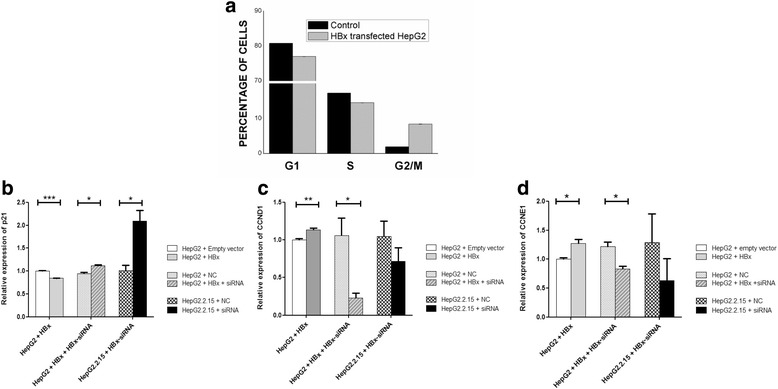
Flow cytometric analysis and alteration of cell cycle regulators upon HBx transfection. **a** Graphical representation of percentage of cells in different phases of cell cycle compared between HepG2 cells transfected with HBx plasmid or empty expression vector as described above. **b**. **c**. **d**. Alteration of cell cycle regulators in HepG2 and HepG2.2.15 cells. Relative mRNA expressions of different cell cycle regulators were compared between HepG2 cells transfected with HBx plasmid and empty vector; X silenced HBx transfected HepG2 cells and negative control; HBx silenced HepG2.2.15 cells and negative control. **b** Relative p21 expression (**c**) Relative CCND1 expression (**d**) Relative CCNE1 expression

We assayed the distribution of cells in the different phases of the cell cycle by flow cytometry analysis. The percentage of cells in different phases of the cell cycle was compared between HBx transfected HepG2 cells and cells transfected with empty plasmid (Fig. 5a). Though the proportion of cells was higher in G1 phase compared to S and G2/M phases, we observed that transfection of HBx plasmid in HepG2 cells induced a release from G1/S arrest. An increase in the G2/M phase cell population in HBx transfected HepG2 cells was evident, when compared to cells transfected with empty plasmid. In order to corroborate the impact of HBx protein on the transition between different phases of cell cycle, we further investigated its effect on the expression of cell cycle genes. As mentioned previously, the analysis of HBx transfected HepG2 cells indicated reduced levels of p53 mRNA as well as protein, which is one of the key regulators in the transition from G1 to S phase. In contrast, p53 mRNA and protein were found to increase when X was silenced in HBx transfected HepG2 and HepG2.2.15 cells. Another protein, p21 is known to be an important regulator of cell cycle progression at G1 and S phase and its expression is specifically controlled by p53 protein. Interestingly, in the HBx transfected HepG2 cells, expression of p21 transcripts was significantly lower (*P* < 0.001) as compared to control cells and its expression was increased significantly (*P* < 0.05) in HBx transfected HepG2 and HepG2.2.15 cells when both the cells were treated with HBx siRNA (Fig. 5b). Expression of CCND1 (cyclin D1) remains high during G1 to S phase transition in the cell cycle. Transfection of HepG2 cells with X gene led to significantly increased (*P* < 0.01) expression of CCND1 RNA compared to control cells. Its expression was found to decrease when HBx transfected HepG2 (*P* < 0.05) and HepG2.2.15 cells were treated with HBx – siRNA (Fig. 5c). CCNE1 (Cyclin E), another important regulator of G1/S transition, remained up regulated in HBx transfected HepG2 cells where as it remained down regulated in X silenced HBx-HepG2 cells (*P* < 0.05) and HepG2.2.15 cells (Fig. 5d).

**Fig. 6:**
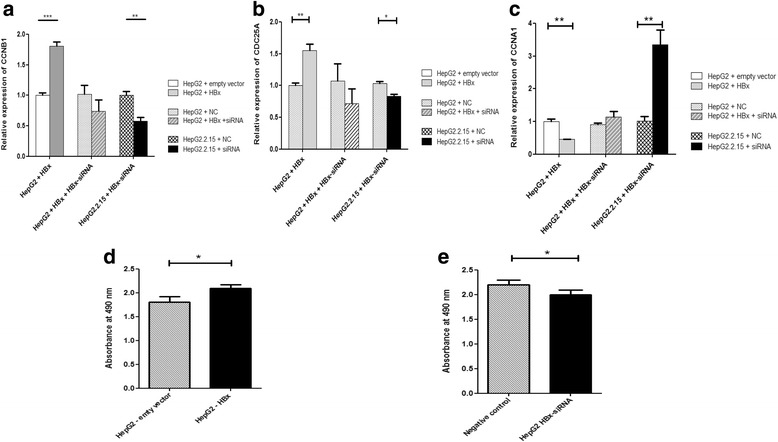
HBx promoted cell proliferation in HepG2 cells. **a**. **b**. **c**. Alteration of G2/M markers in HepG2 and HepG2.2.15 cells. Relative mRNA expressions of different cell cycle regulators were compared between HepG2 cells transfected with HBx plasmid and empty vector; X silenced HBx transfected HepG2 cells and negative control; HBx silenced HepG2.2.15 cells and negative control. **a** Relative CCNB1 expression (**b**) Relative CDC25A expression (**c**) Relative CCNA1 expression. **d**. **e** Cell proliferation assay in HBx transfected HepG2 cells and X silenced HBx transfected HepG2 cells at the 48 h time-point. **d** Results from MTT assay showed that HBx transfection led to a significant increase in HepG2 cell proliferation compared with control cells (**e**). Cell proliferation was decreased in X silenced HBx - HepG2 cells compared to negative control. Data shown represent the mean ± SD. The experiments were performed in quadruplet (*P < 0.05; Student’s *t*-test)

We observed there was a release from G1/S arrest and a marked alteration of cell cycle regulators for G1/S phase in HBx transfected HepG2 cells. Reduction of the relative proportion of cell populations in S phase and visible increase in G2/M phase was also evident in those cells. In order to find out whether the cells were at G2 arrest or at M phase, we assessed the expression of G2/M markers. In HBx transfected HepG2 cells we found significantly increased level of CCNB1 (cyclin B) expression (*P* < 0.001) whereas in HBx silenced HepG2 cells and HepG2.2.15 cells (P < 0.01), its expression was found to be down regulated (Fig. 6a). We also examined another G2/M marker, CDC25A, a member of CDC25 family of M phase inducer phosphatase. In HBx transfected HepG2 cells a significantly increased level of CDC25A expression (*P* < 0.01) was observed compared to control cells. CDC25A expression was found to decrease in HBx- HepG2 cells and HepG2.2.15 cells (*P* < 0.05) when these cells were treated with HBx-siRNA (Fig. 6b). Apart from CCNB1 and CDC25A, we also studied the expression profile of CCNA1 (cyclin A), which acts on G2/M transition, in addition to its regulation of the S phase. We noticed decreased expression of CCNA1 in HBx transfected HepG2 cells (*P* < 0.01) and increased expression of CCNA1 in X silenced HBx- HepG2 cells and HepG2.2.15 cells (*P* < 0.01) (Fig. 6c). This reduced expression of CCNA1 is consistent with high expression of CCNB1- the mitotic marker, as observed in HBx transfected HepG2 cells.

Further we performed an MTT assay to measure the cell proliferation. After 48 h of transfection, HepG2 cells transfected with HBx plasmid exhibited significant (*P* < 0.05) cell proliferation compared to its control cell (Fig. 6d). Interestingly, when HepG2 cells were co transfected with HBx plasmid and HBx siRNA, we observed the reverse scenario i.e. cell proliferation was reduced in HBx silenced HepG2 cells than its negative control (Fig. 6e).

## Discussion

Hepatitis B virus X protein is often referred to as an onco protein as it interacts and modulates the expression of cellular genes, which in turn alter the cell signaling and other cellular processes. HBx induces persistent changes in different cellular genes that subsequently provide a signal to hepatocytes for growth and proliferation thus leading to the development of hepatocellular carcinoma [29–32]. Involvement of microRNA is being uncovered in almost all aspects of cancer biology, such as proliferation, tumorigenesis, apoptosis, invasion/metastasis and angiogenesis [33, 34]. The expression of tumor suppressor miR- 122 is often found down regulated in hepatocellular carcinoma and chronic HBV patients as also in HCC derived cell lines. Our study demonstrated involvement of HBx protein in the modulation of miR-122 expression in hepatoblastoma cell lines. We have taken CCNG1 as the reported target of miR- 122.

It has been documented that miR-122 is abundant in liver and is characteristically down regulated in 70 % of HCCs [18, 35]. Our study on HBV infected patients with different clinical outcomes (advanced liver disease patients or its subset LC and HCC patients) demonstrated that miR-122 expression was decreased, as compared to healthy controls. As mentioned, CCNG1 is the target of miR-122 and an inverse relation exists between them in HCC derived cell lines and HCC tissues. CCNG1 is the only known cyclin that bears two functional binding sites for p53 tumor suppressor protein and is transcriptionally triggered by this transcription factor. Several groups have reported that HBx proteins interact directly with p53 as the binding region of p53 is located within the transactivation domain of HBx protein [5]. In transgenic mice that express HBx, tumor development correlates precisely with the binding of p53 to HBx in the cytoplasm and causes complete impediment of the translocation of p53 to the nucleus [36]. miR-122 was shown to increase p53 protein levels and activity through its negative regulation of cyclin G1 [16]. Cyclin G1 forms a complex with PP2A phosphatase and enhances MDM2 activity to inhibit p53 [37]. By directly repressing the expression of CCNG1, miR-122 increases p53 protein levels and activity and inhibits tumorigenesis in liver cancer. We observed miR-122 expression is reduced and cyclin G1 protein expression is elevated in HBx transfected HepG2 cells. We also noticed p53 mRNA and protein levels were decreased in HBx transfected HepG2 cells. Conversely, silencing of HBx mRNA in HBx transfected HepG2 and HepG2.2.15 cells revealed an increase in p53 mRNA and protein expression. In this light, our results suggest that HBx protein caused down regulation of miR-122 that induce up regulation of CCNG1 in HepG2 cells which produce hindrance to the activity of p53 protein. In addition, reduced level of miR-122 and elevated level of CCNG1expression was noted in HBV genome transfected HepG2 cells and in HepG2.2.15 cells. Moreover, HBx siRNA treatment in transiently transfected HepG2 cells and in HepG2.2.15 cells rescued miR-122 expression.

Our data suggested that transfection of HBx in HepG2 cells suppressed miR-122 expression which in-turn induced increased expression of cyclin G1. Involvement of cyclin G1 in cellular growth control still remains controversial, however it has been observed to be involved in the G2/M arrest in response to DNA damage, suggesting its growth inhibitory activity [38]. On the other hand, several reports indicate a cellular growth promoting activity for cyclin G1, since overexpressed cyclin G1 increases cell growth of cancer cells [39] and its silencing reduces cell proliferation [40]. Increased levels of CCNG1 were found in several human tumours such as breast cancer and osteosarcoma [41, 42]. In the present study, flow cytometry analysis revealed that transfection of HBx plasmid in HepG2 cells induced a release from G1/S arrest. We also found several markers for G1/S phase transition to be modulated in these cells. Interestingly, several lines of evidence have demonstrated that HBx accelerated the release of cells from G0/G1 and their entry into S phase by enabling a rapid activation of CDK2 kinase activity [43]. HBx mediated stimulation of Src kinases and activation of cyclin A- CDK2 complexes was found to force growth-arrested cells to transit through G1 phase [29].

We found increased population of HBx transfected HepG2 cells to be in the G2/M phase by flow cytometry analysis. Whether cells were at G2 arrest or at mitotic divisional phase sorted out by studying G2/M markers and cell proliferation assay, which revealed HBx transfected HepG2 cells are in active divisional phase and this ectopic expression of HBx induced proliferation of these cells. In line with previous reports supporting the growth promoting function of CCNG1, our study demonstrated that HBx evoked increased CCNG1 expression as a result of miR-122 suppression which enhanced proliferation of HepG2 cells.

**Fig. 7:**
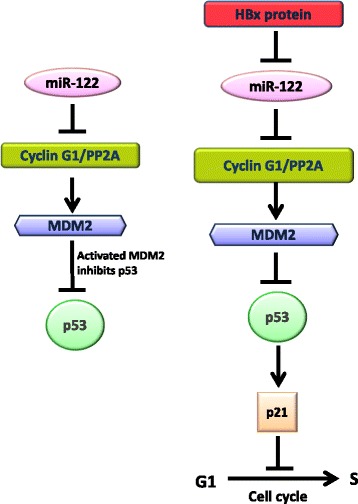
Schematic illustration describing HBx mediated miR-122 suppression enhances hepatoblastoma cell proliferation through cyclin G1/p53 axis. Normally miR-122 enhances p53 activity through down-regulation of its target protein cyclin G1 that forms a complex with PP2A phosphatase and enhances MDM2 activity to inhibit p53. HBx protein of HBV induces down regulation of miR-122 which up regulates cyclin G1 expression. Since cyclin G1 is a negative regulator of p53, up regulated cyclin G1 reduces the expression of p53. As a result expression of p21, which is specifically modulated by p53, is also reduced. Reduced expression of p21 releases the cells from G1 arrest thus facilitating cells for proliferation

Li et al. [44] demonstrated that all four HBV mRNAs bear a complimentary miR-122 sequence which act as sponges to bind and sequester endogenous miR-122. This study suggested HBV transcripts are involved in HBV-mediated miR-122 suppression. Very recently Liang et al. [45] showed that HBx-LINE1, a hybrid RNA transcript originating from human LINE1 and the HBV X gene, consists of six miR-122-binding sites and enforced expression of HBx-LINE1 successfully exhausted cellular miR-122 expression thereby promoting mouse hepatic injury and EMT-like changes in liver cell. Development of hepatic cancer is a multistep process and involves multifactorial etiology. Apart from modulation of a number of cell signaling pathways, different genetic and epigenetic factors are altered in HBV associated hepatocellular carcinoma. A previous study by Song et al. [46] established that HBx binds to PPARc and prevents the transcription of miR-122. Peng et al. [47] revealed that reduction of Gld2 level mediated by HBx resulted in down-regulation of miR-122. Multiple regulatory mechanisms may coexist in HBV-infected cells that control miR-122 levels. Our study provided evidence of HBx-miR-122- CCNG1/p53 mediated induction of cell proliferation that may culminate into hepatic cancer (Fig. 7).

## Conclusions

In conclusion, our study indicated that the HBx protein of HBV induced down regulation of highly abundant liver specific miR-122 in hepatoblastoma cells. HBx mediated suppression of miR-122 enhanced cell proliferation of HepG2 through cyclin G1-p53 mediated pathway. The outcome of this study expands our knowledge about complex host viral interactions leading to alteration of cellular genes, culminating in hepatic proliferation. Furthermore, this study also provides a potential therapeutic target for utilizing the HBx - miR-122 interaction as an effective strategy for management of HBV related HCC.

## Abbreviations

AP, Activator protein; ATF, Activating transcription factor; CDK, Cyclin depandant kinase; CREB, cAMP response element-binding protein; DNA, De oxy ribo nucleic acid; HBV, Hepatitis B virus; HBx, Hepatitis B virus X protein; HCC, Hepatocellular carcinoma; JAK-STAT, Janus kinase-Signal transducer and activator of transcription; LC, Liver cirrhosis; MAP3K, Mitogen activated protein kinase kinase kinase; MDM2, Mouse double minute 2 homologue; miRNA, MicroRNA (Ribo nucleic acid); PBS, phosphate buffer saline; PI3K, Phosphatidylinositide 3-kinase; PP2A, Protein phosphatase 2; PTEN, Phosphate and tensin homologue; RNA, Ribo nucleic acid; RT-PCR, Real time polymerase chain reaction; TBP, TATA binding protein; TERT, Telomerase reverse transcriptase; TNF, Tumour necrosis factor.
